# Designing web-apps for smartphones can be easy as making slideshow presentations

**DOI:** 10.1186/1756-0500-7-94

**Published:** 2014-02-20

**Authors:** Yousif Subhi, Tobias Todsen, Charlotte Ringsted, Lars Konge

**Affiliations:** 1 The Centre for Clinical Education, University of Copenhagen and the Capital Region of Denmark, Copenhagen, Denmark; 2 Department of Ophthalmology, Copenhagen University Hospital Roskilde, Roskilde, Denmark; 3 Department of Anesthesia, The Wilson Centre, University of Toronto and The University Health Network, Toronto, Canada

**Keywords:** Smartphones, Apps, Clinician involvement

## Abstract

**Background:**

Limited clinician involvement in smartphone application development poses problems considering the extensive use of smartphones among medical professionals and patients.

**Findings:**

We present a simple method for the clinician to develop simple web-apps using only an Internet browser and a text editor.

**Conclusions:**

This method may help clinicians develop simple web-apps and increase clinician involvement in smartphone content.

## Findings

The impressive distribution and use of smartphones among health professionals and patients promise interesting advances within fields such as medical education, patient education, patient aid, and telemedicine [1-3]. However, anyone can publish software applications for smartphones (apps) without clinician involvement. Existing apps should be carefully reviewed before any recommendation as the only restriction before publication is $99/year [4,5]. Many existing apps are of low quality and some are even unsafe [5,6]. There is a need for increased clinician involvement in the development of smartphone apps [7,8].

Clinicians diligently develop digital and printed material, but may be reluctant from developing apps as required technical skills or resources may be insufficient. While some smartphone apps may require advanced algorithms or technology, many popular apps are actually quite simple and consist only of text and images. In fact, simple web-apps — smartphone applications that function using the build-in web browser — can be developed as easy as preparing slideshows. Here, we describe one easy method to develop a simple web-app by only using an Internet browser and a text editor.

In general, the design consists of several different frames, of which only one is visible to the user. The user will start at the main frame, and then navigate to other frames. Each of these frames and the desired connections can be designed using the drag-and-drop user interface on the jQuery Mobile website [9] based on a Codiqa interface [10]. This interface generates codes corresponding to the designed content. These codes can be collected for each frame and then put together in a single text-file. Finally, two code-snips [see Additional file 1 & Additional file 2] should be copy-pasted to the top and the bottom of the text-file respectively for the web-app to function. The final text-file should be saved as a webpage, i.e. with .html as the filename-extension (e.g. mywebapp.html), and then uploaded to a webserver from which the users may access the web-app. We have developed an instructional video to demonstrate our method and how a web-app looks on the iPhone [see Additional file 3]. Our example web-app developed in the video is also available [see Additional file 4].

Two limitations of this method should be noted. Firstly, programming assistance may be needed for enabling more advanced functionalities. Secondly, web-apps are not indexed in iTunes App Store or Google Play, and therefore users will need a link to access the web-app, unless it is converted to native smartphone apps using conversion tools, such as PhoneGap [11] or Codiqa [10]. The web-app is then converted to a format compatible with iTunes App Store and Google Play, and distributed to the common user. The herein described method may help clinicians develop simple web-apps and thereby open the door for a mean of communication, which only seems to increase in importance [1]. However, even with the involvement from clinicians, anything read on the Internet should be critically read, and can contain errors or be potentially dangerous even with relevantly involved clinicians.

## Availability and requirements

**Project name:** Designing web-apps for smartphones can be easy as making slideshow presentations

**Project home page:** None.

**Operating system(s):** Platform independent

**Programming language:** HTML, CSS, and JS

**Other requirements:** A simple text-editing program, e.g. Notepad

**Any restrictions to use by non-academics:** None (MIT License)

### Availability of supporting data

The data sets supporting the results of this article are included within the article and its additional files.

## Competing interests

Yousif Subhi, Tobias Todsen, Charlotte Ringsted, and Lars Konge have no conflicts of interests to declare.

## Authors’ contributions

YS contributed with the original idea and drafted the manuscript. YS and TT recorded the instructional video. TT, CR, and LK helped to draft the final manuscript. All authors read and approved the final manuscript.

## Authors’ information

YS is medical graduate student and TT is medical doctor at the University of Copenhagen and work at the Centre for Clinical Education at the University of Copenhagen and the Capital Region of Denmark investigating how smartphones are used in medical education. YS is also a research assistant at the Department of Ophthalmology at the Copenhagen University Hospital Roskilde. CR is director and scientist of the Wilson Centre and professor in the Department of Anesthesia at the University of Toronto. LK is clinical associate professor and senior consultant at the Centre for Clinical Education at the University of Copenhagen and the Capital Region of Denmark.

## Supplementary Material

Additional file 1 **Code-snip top.** Description: Code-snip to be inserted at the top of the text-file. Click here for file

Additional file 2 **Code-snip bottom.** Description: Code-snip to be inserted at the bottom of the text-file. Click here for file

Additional file 3 **Instructional video.** Description: Instructional video on how to develop a simple web-app. Click here for file

Additional file 4 **Example web-app.** Description: The example web-app developed in the instructional video. Click here for file
