# Supplementary material for: Designing web-apps for smartphones can be easy as making slideshow presentations
Source: BMC Res Notes. 2014 Feb 20;7:94. doi: 10.1186/1756-0500-7-94 (PMC3931664; doi:10.1186/1756-0500-7-94)
Supplement: Additional file 4 — Example web-app. Description: The example web-app developed in the instructional video. [file 1756-0500-7-94-S4.html]

### Retina

##### The retina is the photo-receptive tissue lining the inner surface of the eye, upon which the optics of the eye focuses the images.

##### The retina has two types of photo-receptors and is composed of several layers.

Retinal structure

Back

### Retinal structure

##### The retina is composed of several layers:
